# Dentin sialoprotein promotes endothelial differentiation of dental pulp stem cells through DSP_aa34–50_–endoglin–AKT1 axis

**DOI:** 10.1016/j.jbc.2025.108380

**Published:** 2025-03-04

**Authors:** Ximin Xu, Jing Fu, Guobin Yang, Zhi Chen, Shuo Chen, Guohua Yuan

**Affiliations:** 1 State Key Laboratory of Oral & Maxillofacial Reconstruction and Regeneration, Key Laboratory of Oral Biomedicine Ministry of Education, Hubei Key Laboratory of Stomatology, School & Hospital of Stomatology, Wuhan University, Wuhan, Hubei, China; 2 Frontier Science Center for Immunology and Metabolism, Wuhan University, Wuhan, China; 3 Hubei Provincial Key Laboratory of Developmentally Originated Disease, Wuhan, Hubei, China; 4 Department of Developmental Dentistry, School of Dentistry, The University of Texas Health Science Center at San Antonio, San Antonio, Texas, United States

**Keywords:** dentin sialoprotein, endoglin, endothelial differentiation, AKT1, dental pulp stem cells

## Abstract

Dentin sialoprotein (DSP), a major dentin extracellular matrix noncollagenous protein, is well recognized as an important regulator for dentinogenesis. DSP as a secreted protein can interact with membrane receptors, activate intracellular signaling, and initiate the odontoblastic differentiation of dental papilla cells. In a recent study, we have demonstrated that DSP can induce the endothelial differentiation of dental pulp stem cells (DPSCs), a type of tooth pulp–derived multipotent stem cells, dependent on membrane receptor endoglin (ENG). However, the intimate mechanisms by which DSP–ENG association facilitates the endothelial differentiation of DPSCs remain enigmatic. Here, we find that the amino acid (aa) residues 34–50 of DSP (DSP_aa34–50_) is responsible for its association with ENG using a series of co-immunoprecipitation assays. Immunofluorescent staining and *in situ* proximity ligation assay demonstrate that overexpressed ENG in human embryonic kidney 293T cells shows codistribution and proximity ligation assay signals to the supplemented DSP_aa34–50_ protein but not to DSP without aa34–50 (DSP_Δ34–50_) on cell surfaces. Moreover, the zona pellucida domain of ENG mediates its association with DSP_aa34–50_. Further experiments indicate that DSP_aa34–50_ exhibits equivalent effects to the full-length DSP on the migration and endothelial differentiation of DPSCs dependent on ENG but DSP_Δ34–50_ does not. Mechanistically, DSP_aa34–50_ activates AKT1 and triggers the expression of blood vessel development–related genes in DPSCs. Multiple experiments demonstrate that AKT1 inhibition suppresses the DSP_aa34–50_-induced migration and endothelial differentiation of DPSCs. Thus, AKT1 mediates the cellular and molecular functions of DSP_aa34–50_–ENG association. Collectively, these findings identify that DSP promotes the endothelial differentiation of DPSCs through the DSP_aa34–50_–ENG–AKT1 signaling axis.

Dentin sialoprotein (DSP) is a secreted noncollagenous protein essential for dentin formation during tooth development (1, 2, 3, 4). It is the NH2-terminal cleavage product of dentin sialophosphoprotein (DSPP), which is mainly expressed and synthesized by odontoblasts in the tooth, and DSP is further proteolytically cleaved into small fragments (5, 6, 7, 8). Previous *in vitro* cell culture systems and *in vivo* models have demonstrated that DSP and its peptides can bind to the membrane receptors, like integrin β6 and Ocluddin (Ocln), to facilitate intracellular signaling, and thereby regulating the odontoblastic differentiation and mineralization ability of dental mesenchymal cells (9, 10).

In addition to involvement in dentinogenesis, several clues indicate that DSP may also regulate angiogenic processes. First, DSP-coated agarose beads induce blood vessel formation and invasion in reparative dentin regeneration experiments (9). Second, the presence of DSP in teeth starts from embryonic day (E) 17, which is concomitant with fast vascular formation and remodeling in the dental mesenchyme (11, 12, 13). Third, *Dspp* deficient mice exhibit reduced blood vessel formation in the transition zone of femurs (14, 15). In addition, our recent study has identified that DSP facilitates the endothelial differentiation of dental pulp stem cells (DPSCs).

DPSCs are a subpopulation of tooth-derived mesenchymal stem cells (MSCs) with multilineage differentiation potentials (16, 17). In addition to odontoblasts, osteoblasts, chondrocytes, and adipocytes, DPSCs have been reported to be able to differentiate into endothelial cells (18, 19, 20, 21). Growth factors, like vascular endothelial growth factor (VEGF), and several signaling axes, such as Wnt/β-catenin, PI3K–AKT, and MER–ERK1/2, have been reported to regulate the endothelial differentiation of DPSCs (22, 23, 24, 25, 26, 27). As a type of MSCs, DPSCs show high expression level of endoglin (ENG), and a previous study has demonstrated that ENG-positive DPSCs show high angiogenic potential (21). Besides, our recent work identifies that ENG mediates the DSP-induced endothelial differentiation of DPSCs. As a secreted protein, the domains of DSP that are responsible for its association with other membrane receptors, integrin β6 and Ocln, have been reported (10, 11). However, the specific fragment of DSP that associates with ENG and the downstream signaling pathway activated by DSP–ENG association remain to be elucidated.

In this study, we find that the DSP domain containing the amino acid (aa) residues 34–50 (DSP_aa34–50_) and the zona pellucida (ZP) domain of ENG mediate their association. The effects of DSP_aa34–50_ on the migration and endothelial differentiation of DPSCs are identical to the full-length protein. Mechanistically, AKT1 signaling is activated by DSP_aa34–50_–ENG association, and AKT1 inhibition or *ENG* knockdown inhibits the endothelial differentiation of DPSCs induced by DSP. Thus, our study unveils that AKT1 signaling mediates the endothelial differentiation of DPSCs triggered by DSP_aa34–50_–ENG association.

## Results

### DSP associates with ENG through amino acid residues 34–50 of DSP

**Figure 1:**
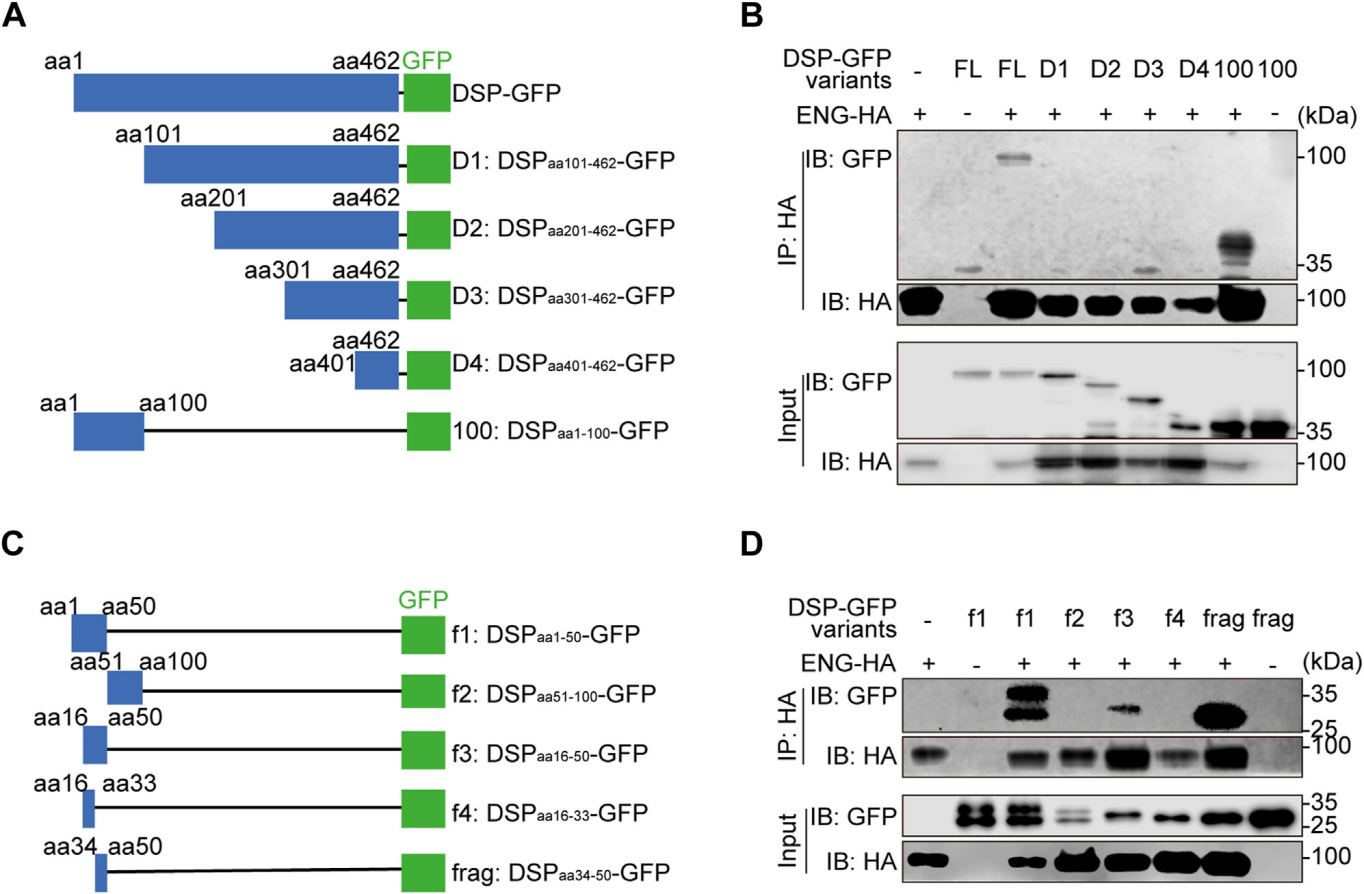
**DSP**_**aa34–50**_**is associated with ENG.***A* and *C*, schematic diagrams of plasmids encoding GFP-tagged full-length and truncated DSPs. *B* and *D*, co-IP assays demonstrated that the DSP_aa34–50_ was co-immunoprecipitated with ENG. The data are representatives of three independent experiments, respectively. Co-IP, co-immunoprecipitation; D, delete; DSP, dentin sialoprotein; ENG, endoglin; f, fragment; FL, full length.

**Figure 2:**
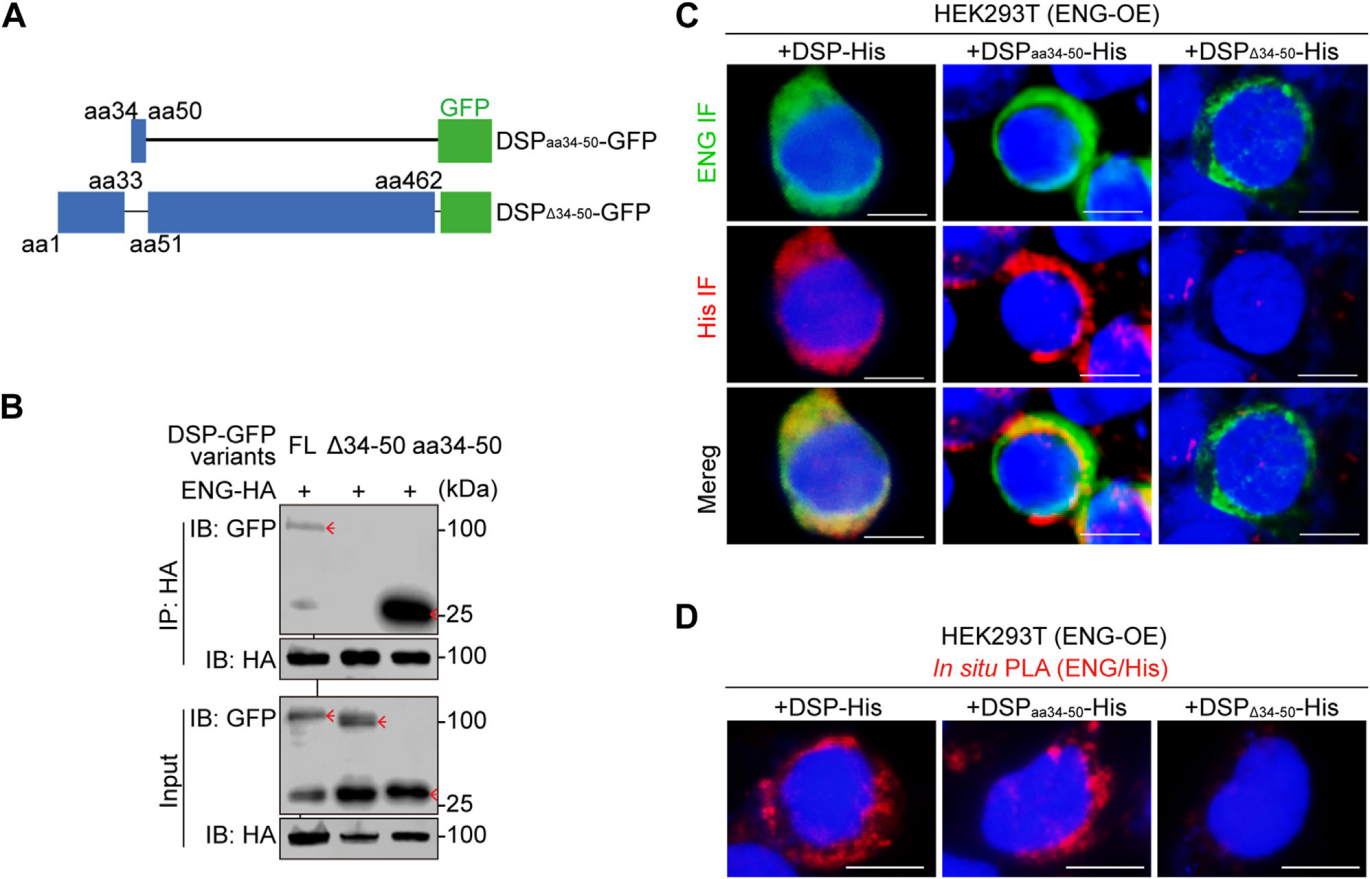
**The amino acid 34**–**50 of DSP is responsible for DSP–ENG association.***A*, schematic diagrams of plasmids encoding GFP-tagged truncated DSP mutants. *B*, co-IP assays revealed that DSP_aa34–50_ was responsible for DSP–ENG association. *Red arrows* point to the bands of GFP-tagged DSP, GFP-tagged DSP_aa34–50_, and GFP-tagged DSP_Δ34–50_. The data are representatives of three independent experiments. *C* and *D*, HEK293T cells with ENG overexpression (ENG-OE) were incubated with DSP-His, DSP_aa34–50_-His, or DSP_Δ34–50_-His protein (0.5 μg/ml) at 37 °C for 1 h, and immunofluorescent (IF) staining as well as *in situ* PLA assay were performed. The images were captured using OLYMPUS BX53 fluorescence microscope. Representative IF images of ENG and His in HEK293T (ENG-OE) cells were shown (*C*). Representative images showed the DSP–ENG or DSP_aa34–50_–ENG PLA signals on cell surfaces (*D*). The experiments were performed at least three times. Scale bars represent 20 μm for (*C* and *D*). co-IP, co-immunoprecipitation; DSP, dentin sialoprotein; ENG, endoglin; HEK293T, human embryonic kidney 293T cell line; PLA, proximity ligation assay.

To define the specific domain of DSP that interacts with ENG, plasmids encoding GFP-tagged truncated DSPs were constructed and respectively transfected into human embryonic kidney 293T (HEK293T) cells together with plasmid encoding hemagglutinin (HA)-tagged ENG (Fig. 1*A*). Co-immunoprecipitation results demonstrated that it was the aa1–100 of DSP that was pulled down by ENG, and subsequent experiments further showed that DSP fragment covering aa34–50 was co-immunoprecipitated by ENG (Fig. 1, *B*–*D*). Consistently, deletion of aa34–50 in DSP abolished it (Fig. 2, *A* and *B*), confirming that DSP_aa34–50_ accounted for DSP–ENG association. Then recombinant DSP_aa34–50_ and DSP_Δ34–50_ (DSP with deletion of aa34–50) proteins were purified, and respectively, used to treat HEK293T cells with ENG overexpression. Immunofluorescent staining showed that, similar to the full-length DSP protein, DSP_aa34–50_ was codistributed with ENG on the surfaces of HEK293T cells but DSP_Δ34–50_ was not (Fig. 2*C*). Consistently, *in situ* proximity ligation assay (PLA) demonstrated that DSP_aa34–50_, like the full-length DSP, showed the PLA signals with ENG on the cell surfaces but DSP_Δ34–50_ was not (Fig. 2*D*). In postnatal 2 mouse molars, double immunofluorescent staining showed the codistribution of DSP and ENG on the cell surfaces of dental papilla cells *in vivo* (Fig. S1, *A*–*C*). Meanwhile, *in situ* PLA assays verified that endogenous DSP and ENG were in proximity on the surfaces of dental papilla cells *in vivo* (Fig. S1*D*).

### DSP_aa34–50_ associates with the ZP domain of ENG

**Figure 3:**
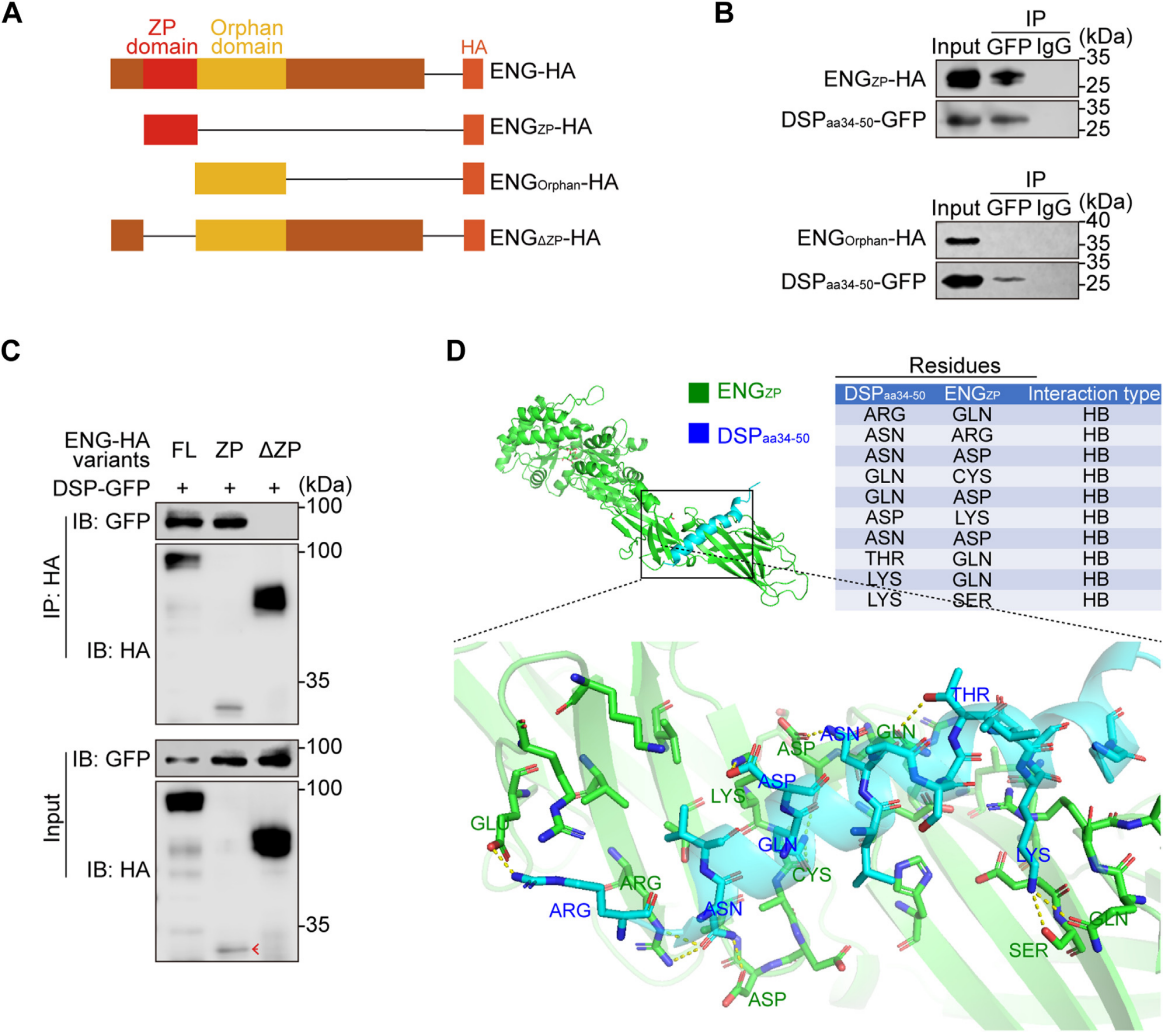
**The ZP domain of ENG accounts for DSP–ENG association.***A*, schematic diagrams of plasmids encoding HA-tagged ENG and truncated mutants. *B* and *C*, co-IP assays demonstrated that the ZP domain of ENG accounts for DSP–ENG association. *Red arrows* point to the bands of HA-tagged ENG_ZP_. Three independent experiments were performed. *D*, molecular docking of DSP_aa34–50_ and ENG_ZP_. Co-IP, co-immunoprecipitation; DSP, dentin sialoprotein; ENG, endoglin; HB, hydrogen bonding; ZP, zona pellucida.

The extracellular part of ENG has two domains, the Orphan domain and the ZP domain (28). To determine which domain of ENG is responsible for its association with DSP, plasmids encoding HA-tagged truncated ENG were constructed (Fig. 3*A*). The ZP domain of ENG was pulled down by DSP_aa34–50_, but the Orphan domain was not (Fig. 3*B*). Truncated ENG with the ZP domain deleted failed to pull down DSP (Fig. 3*C*). Moreover, a three-dimensional structural model showed potential DSP_aa34–50_–ENG_ZP_ interaction (Fig. 3*D*). Therefore, aa34–50 of DSP and the ZP domain of ENG mediate their association.

### DSP_aa34–50_ performs the same effects on DPSCs as the full-length DSP protein dependent on ENG

**Figure 4:**
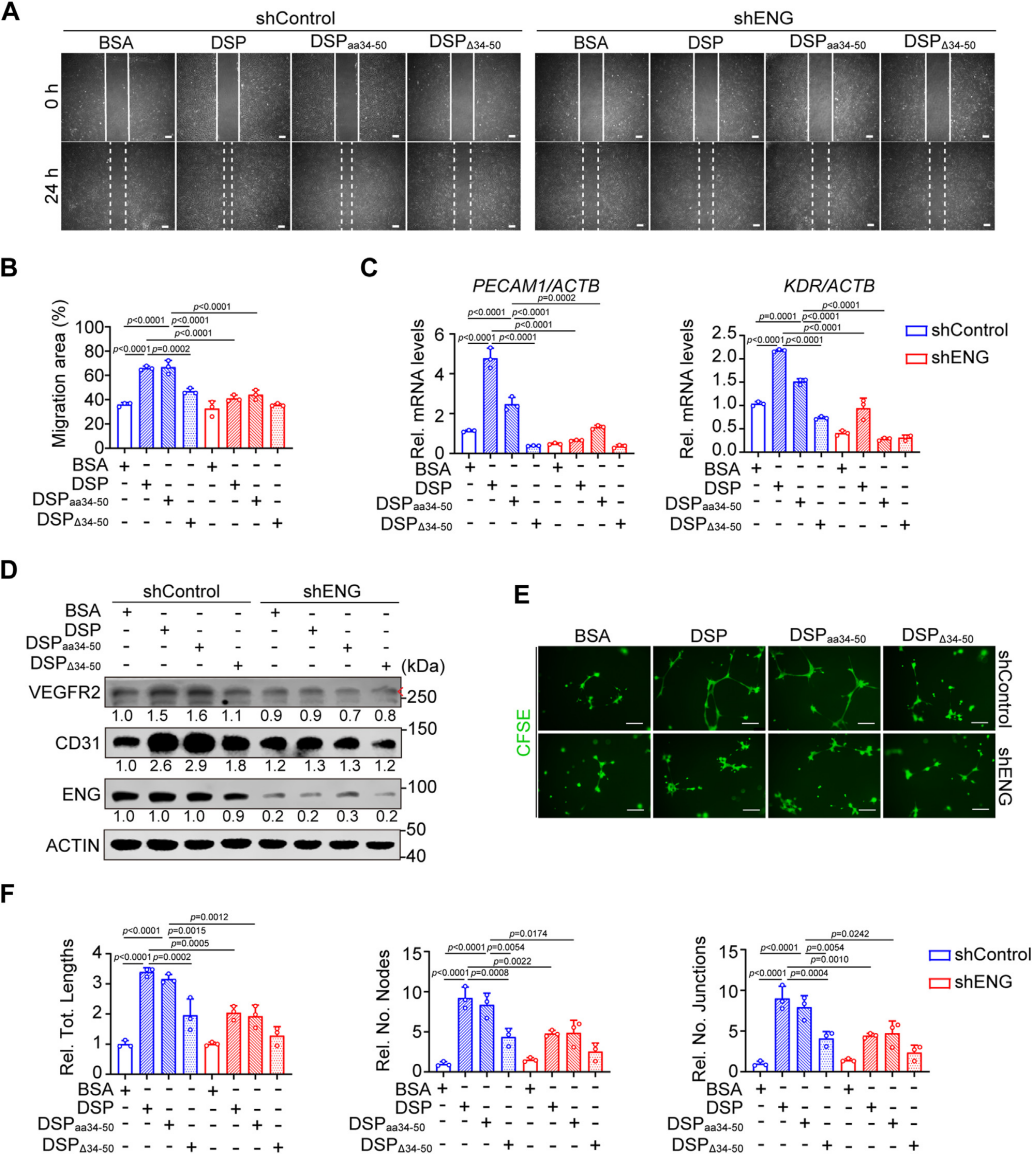
**DSP**_**aa34–50**_**, similar to the full-length DSP protein, promotes the migration and endothelial differentiation of DPSCs dependent on ENG *ex vitro*.** DPSCs with or without *ENG* knockdown were treated with BSA, DSP, DSP_aa34–50,_ or DSP_Δ34–50_ at the concentration of 300 ng/ml. *A*, representative images of scratch wound healing assays in DPSCs with indicated treatment. The leading edges of cells at 0 and 24 h were marked by *solid* and *dashed lines*, respectively. *B*, quantitative analysis of migration areas in (*A*) (n = 3). *C*, RT–qPCR showing the mRNA levels of *PECAM1* and *KDR* in DPSCs with indicated protein treatment for 7 days (n = 3). *ACTB* was used for normalization. The delta–delta Ct method was used for calculation. *D*, WB analysis showing the protein levels of CD31 and VEGFR2 in DPSCs with indicated protein treatment for 7 days. ACTIN served as a loading control, and relative ratios of VEGFR2 *versus* ACTIN, CD31 *versus* ACTIN, and ENG *versus* ACTIN were shown. *Red arrow* points to the bands of VEGFR2. *E*, representative images of Matrigel angiogenesis assays of DPSCs with indicated treatment. After *ENG* knockdown, DPSCs were treated with indicated proteins for 7 days. Then cells were collected and seeded onto Matrigel-coated plates. After incubation for 14 h, cells were stained with CFSE (2 μM). *F*, quantification of the tube lengths, the number of nodes, and the number of junctions in (*E*) (n = 3). The quantification results are represented as mean ± SD (*B*, *C*, and *F*). Two-way ANOVA with Tukey's *post hoc* test (*B*, *C*, and *F*). Scale bars represent 200 μm for (*A* and *E*). BSA, bovine serum albumin; CFSE, carboxyfluorescein succinimidyl ester; DPSC, dental pulp stem cell; DSP, dentin sialoprotein; ENG, endoglin; No., number of; qPCR, quantitative PCR; Rel., relative; Tot, total.; VEGFR2, vascular endothelial growth factor receptor 2; WB, Western blot.

**Figure 5:**
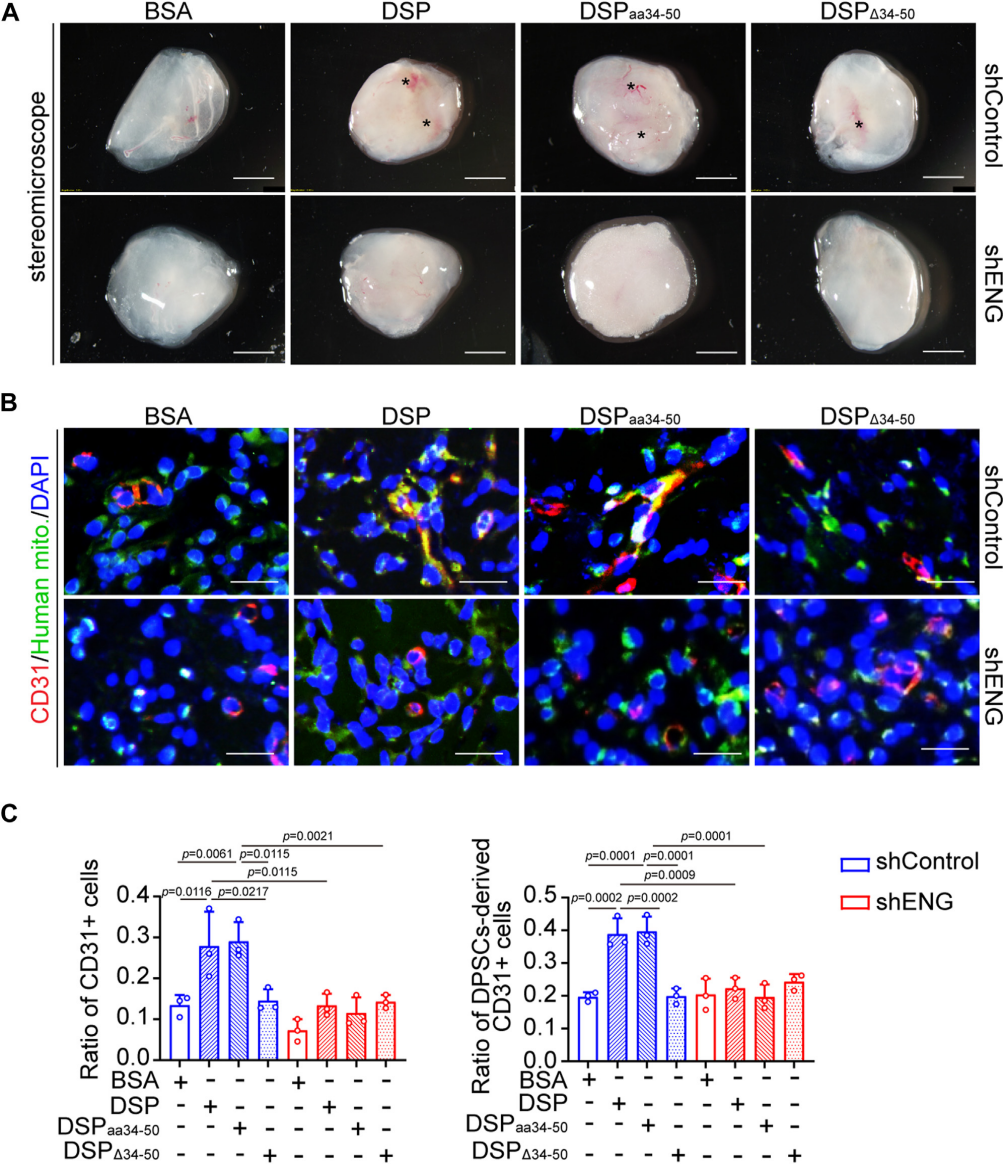
**DSP**_**aa34–50**_**promotes the endothelial differentiation of DPSCs in an ENG-dependent manner *ex vivo*.** DPSCs with or without *ENG* knockdown were treated with indicated proteins (300 ng/ml) for 7 days and then mixed with Matrigel. The mixtures were injected subcutaneously into the ventral side of mice, and the plugs were collected after 7 days. *A*, representative images of Matrigel plugs containing human DPSCs with indicated treatment. *Asterisks* show blood vessels (n = 3). *B*, representative immunofluorescent images of CD31 and human mitochondria in Matrigel plugs containing human DPSCs with indicated treatment. *C*, ratios of CD31+ cells *versus* total cells and DPSC-derived CD31+ cells *versus* total CD31+ cells in (*B*) (n = 3). The quantification results are represented as mean ± SD (*C*). Two-way ANOVA with Tukey's *post hoc* test (*C*). Scale bars represent 2 mm for (*A*) and 50 μm for (*B*). DSP, dentin sialoprotein; DPSC, dental pulp stem cell; ENG, endoglin; Human mito., human mitochondria.

To assess whether DSP_aa34–50_ is the functional fragment of the full-length DSP protein, DPSCs with or without *ENG* knockdown were incubated with bovine serum albumin (BSA), DSP, DSP_aa34–50_, or DSP_Δ34–50_. Scratch wound healing assays demonstrated that DSP_aa34–50_ stimulated the migration of DPSCs comparably to the full-length protein, but DSP_Δ34–50_ did not show this effect (Fig. 4, *A* and *B*). Meanwhile, in contrast to DSP_Δ34–50_, DSP_aa34–50_ elevated the expression of endothelial differentiation markers, including mRNA levels of platelet and endothelial cell adhesion molecule 1 (*PECAM1*) and kinase insert domain receptor (*KDR*) as well as protein levels of CD31 and vascular endothelial growth factor receptor 2 (VEGFR2) in DPSCs (Fig. 4, *C* and *D*). Like DSP, DSP_aa34–50_-treated DPSCs formed more capillary-like structures in Matrigel angiogenesis assays than BSA- or DSP_Δ34–50_-treated cells (Fig. 4, *E* and *F*). Consistently, Matrigel plugs containing DSP- or DSP_aa34–50_-treated DPSCs were imbued with more blood vessels than other groups (Fig. 5*A*). More DPSC-derived CD31+ cells were observed in Matrigel plugs containing DSP- or DSP_aa34–50_-treated DPSCs (Fig. 5, *B* and *C*). However, the aforementioned effects of DSP_aa34–50_ on the migration and endothelial differentiation of DPSCs were suppressed by *ENG* knockdown (Figs. 4 and 5). Hence, these data indicate that DSP_aa34–50_ shows similar effects to DSP on the migration and endothelial differentiation of DPSCs dependent on ENG.

### AKT1 signaling is upregulated by DSP_aa34–50_–ENG association

**Figure 6:**
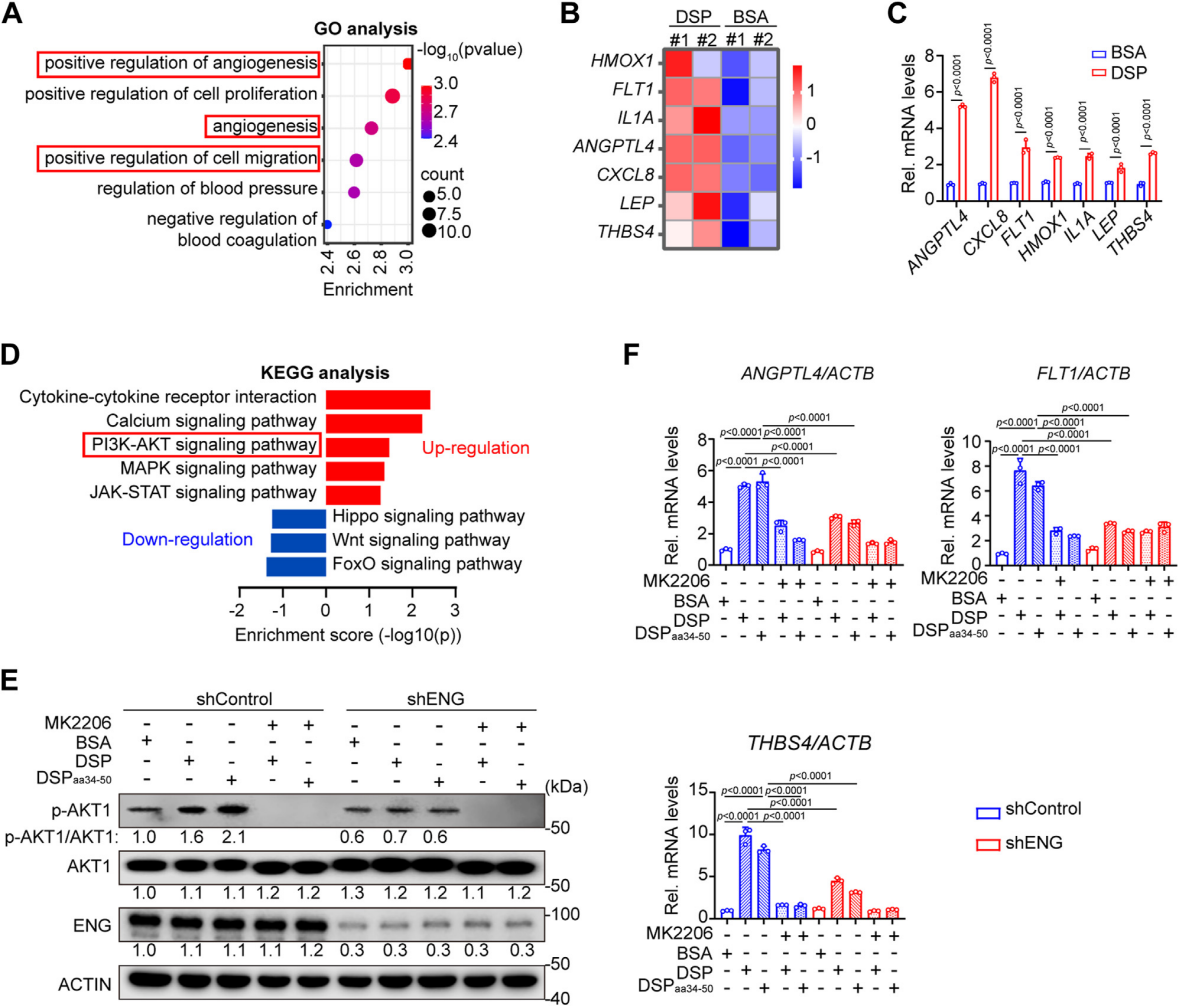
***ANGPTL4*, *FLT1*, and *THBS4* are upregulated by DSP or DSP**_**aa34–50**_**in DPSCs through activating AKT1 signaling.***A*, Gene Ontology (GO) analysis of DSP-treated DPSCs compared with BSA-treated cells. *B*, heatmap of indicated genes in DSP-treated DPSCs compared with BSA-treated cells. *C*, the mRNA levels of indicated genes in DPSCs treated by BSA or DSP (300 ng/ml) by RT–qPCR analysis (n = 3). *ACTB* was used for normalization. The calculation method was delta–delta Ct. *D*, Kyoto Encyclopedia of Genes and Genomes (KEGG) analysis showed the upregulated and downregulated signaling pathways in DSP-treated DPSCs compared with control cells. *E* and *F*, DPSCs with or without *ENG* knockdown or pretreated with MK2206 (2 μM, an inhibitor targeting AKT signaling pathway) or not were cultured with indicated proteins (300 ng/ml). The level of the phosphorylated AKT1 (p-AKT1) in DPSCs was shown by WB analysis. MK2206 is a molecular inhibitor of the AKT pathway. ACTIN served as a loading control, and the relative ratios of p-AKT1 *versus* AKT1, AKT1 *versus* ACTIN, and ENG *versus* ACTIN were shown (*E*). The mRNA levels of *ANGPTL4*, *FLT1*, and *THBS4* in DPSCs shown by RT–qPCR analysis (n = 3). *ACTB* was used for normalization. The calculation method was delta–delta Ct (*F*). The quantification results are represented as mean ± SD (*C* and *F*). Student's *t* test for (*C*) and two-way ANOVA with Tukey's *post hoc* test for (*F*). BSA, bovine serum albumin; DSP, dentin sialoprotein; DPSC, dental pulp stem cell; qPCR, quantitative PCR; WB, Western blot.

To uncover the downstream signaling pathway that mediates the migration and endothelial differentiation of DPSCs triggered by DSP–ENG association, BSA- and DSP-treated DPSCs were harvested for RNA-Seq. Gene Ontology analysis showed that the upregulated genes in DSP-treated cells were enriched in angiogenesis and cell migration (Fig. 6*A*). Several angiogenesis-related genes (*HMOX1*, *FLT1*, *IL1A*, *ANGPTL4*, *CXCL8*, *LEP*, and *THBS4*) were upregulated by DSP, which was further confirmed by real-time quantitative PCR (RT–qPCR) analysis (Fig. 6, *B* and *C*).

Kyoto Encyclopedia of Genes and Genomes analysis demonstrated that several signaling pathways were upregulated (Fig. 6*D*). We focused on the PI3K–AKT signaling pathway because it has been reported to be responsible for the endothelial differentiation of cardiac and adipose-derived stem cells as well as the migration of MSCs (29, 30, 31, 32). Among three different AKT isoforms, AKT1 is ubiquitously expressed, whereas AKT2 is predominantly expressed in insulin-responsive cells and AKT3 is limited in neurons and testes of mice (33). AKT1 was therefore detected in our following experiments. Western blot (WB) analysis showed that the phosphorylation level of AKT1 was enhanced in DSP- and DSP_aa34–50_-treated DPSCs, which was blocked by *ENG* knockdown (Fig. 6*E*). Among the upregulated angiogenesis-related genes, *ANGPTL4*, *FLT1*, and *THBS4* have been reported to be the downstream targets of PI3K–AKT pathway (34, 35, 36). In the presence of MK2206, a pan-inhibitor of AKT (37), the increased mRNA levels of these three genes as well as the upregulated protein level of phosphorylated AKT1 (p-AKT1) were abolished (Fig. 6, *E* and *F*).

### AKT1 signaling mediates the functions of DSP and DSP_aa34–50_

**Figure 7:**
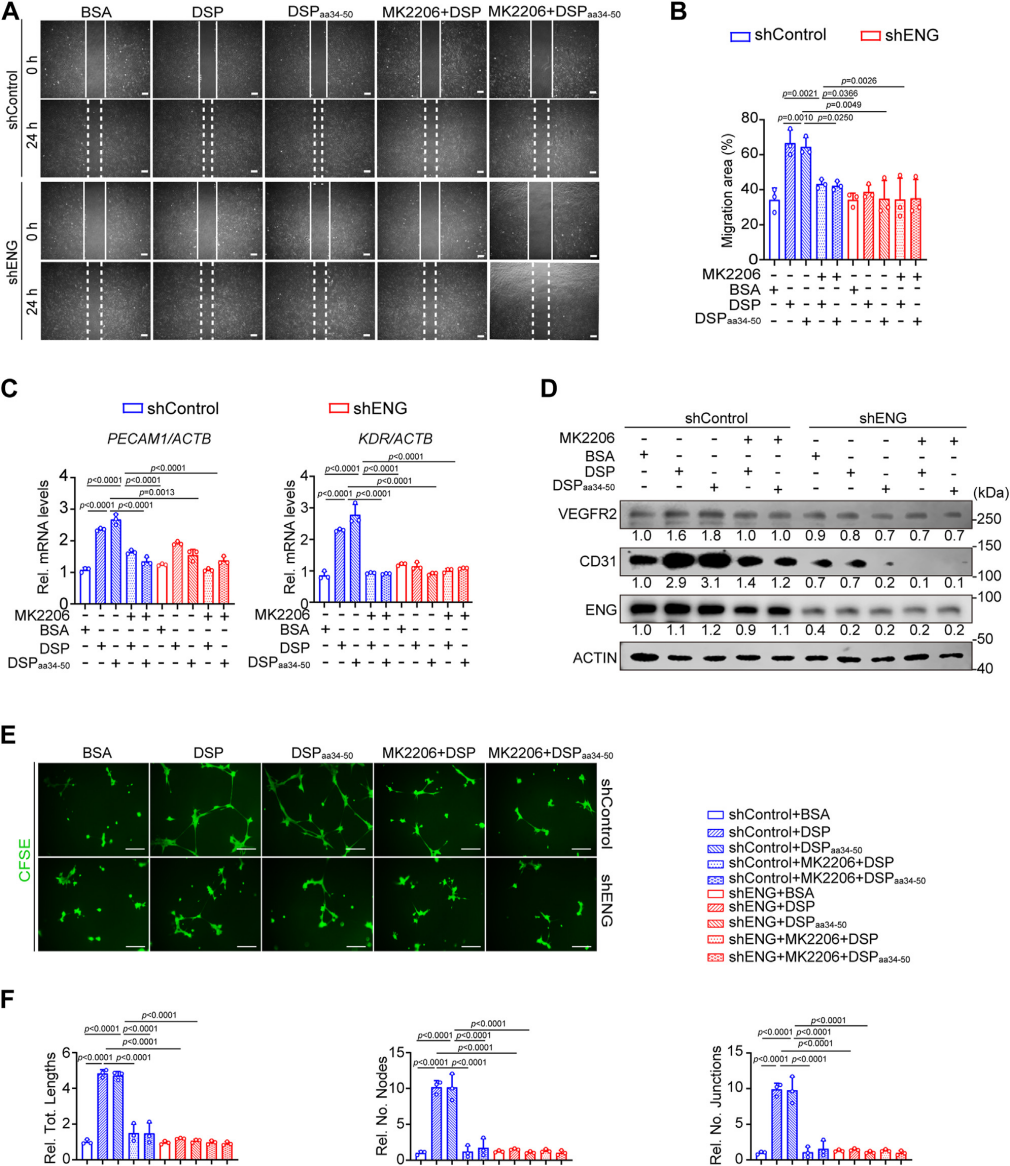
**AKT1 inhibition suppresses the DSP- and DSP**_**aa34–50**_**-induced migration and endothelial differentiation of DPSCs *in vitro*.** DPSCs with or without *ENG* knockdown or pretreated with MK2206 (2 μM) or not were cultured with indicated proteins (300 ng/ml). *A*, representative images of scratch wound healing assays in DPSCs with indicated treatment. The leading edges of cells at 0 and 24 h were marked by *solid lines* and *dashed lines*, respectively. *B*, quantitative analysis of migration areas in (*A*) (n = 3). *C*, RT–qPCR analysis showing the mRNA levels of *PECAM1* and *KDR* in DPSCs with indicated protein treatment for 7 days (n = 3). *ACTB* was used for normalization. The calculation method was delta–delta Ct. *D*, WB analysis showing the protein levels of CD31 and VEGFR2 in DPSCs with indicated protein treatment for 7 days. The *red arrow* points to the bands of VEGFR2. ACTIN served as a loading control. Relative ratios of VEGFR2 *versus* ACTIN and CD31 *versus* ACTIN were shown. *E*, representative images of Matrigel angiogenesis assays of DPSCs. Cells with indicated protein treatment for 7 days were collected and seeded onto Matrigel-coated plates. After incubation for 14 h, cells were stained with CFSE (2 μM). *F*, quantification of the tube lengths, the number of nodes, and the number of junctions in (*E*) (n = 3). The quantification results are represented as mean ± SD (*B*, *C*, and *F*). Two-way ANOVA with Tukey's *post hoc* test (*B*, *C*, and *F*). Scale bars represent 200 μm (*A* and *E*). CFSE, carboxyfluorescein succinimidyl ester; DSP, dentin sialoprotein; DPSC, dental pulp stem cell; ENG, endoglin; qPCR, quantitative PCR; VEGFR2, vascular endothelial growth factor receptor 2.

To determine whether p-AKT1 mediates the effects of DSP–ENG association, DPSCs were pretreated with MK2206 followed by incubation with the presence of DSP or DSP_aa34–50_. MK2206 pretreatment suppressed the enhanced migration of DPSCs because of DSP or DSP_aa34–50_ treatment (Fig. 7, *A* and *B*). Moreover, the elevated *PECAM1* and *KDR* mRNAs as well as the increased CD31 and VEGFR2 proteins facilitated by DSP and DSP_aa34–50_ were reversed by AKT1 inhibition (Fig. 7, *C* and *D*). Considering that MK2206 is a pan-AKT inhibitor (37), siRNAs targeting *AKT1* (siAKT1#1 and siAKT1#2) were used to determine whether AKT inhibition showed the equivalent effects to *AKT1* knockdown. The results demonstrated that both AKT inhibition and *AKT1* knockdown suppressed the upregulated endothelial markers in DPSCs induced by DSP (Fig. S2).

**Figure 8:**
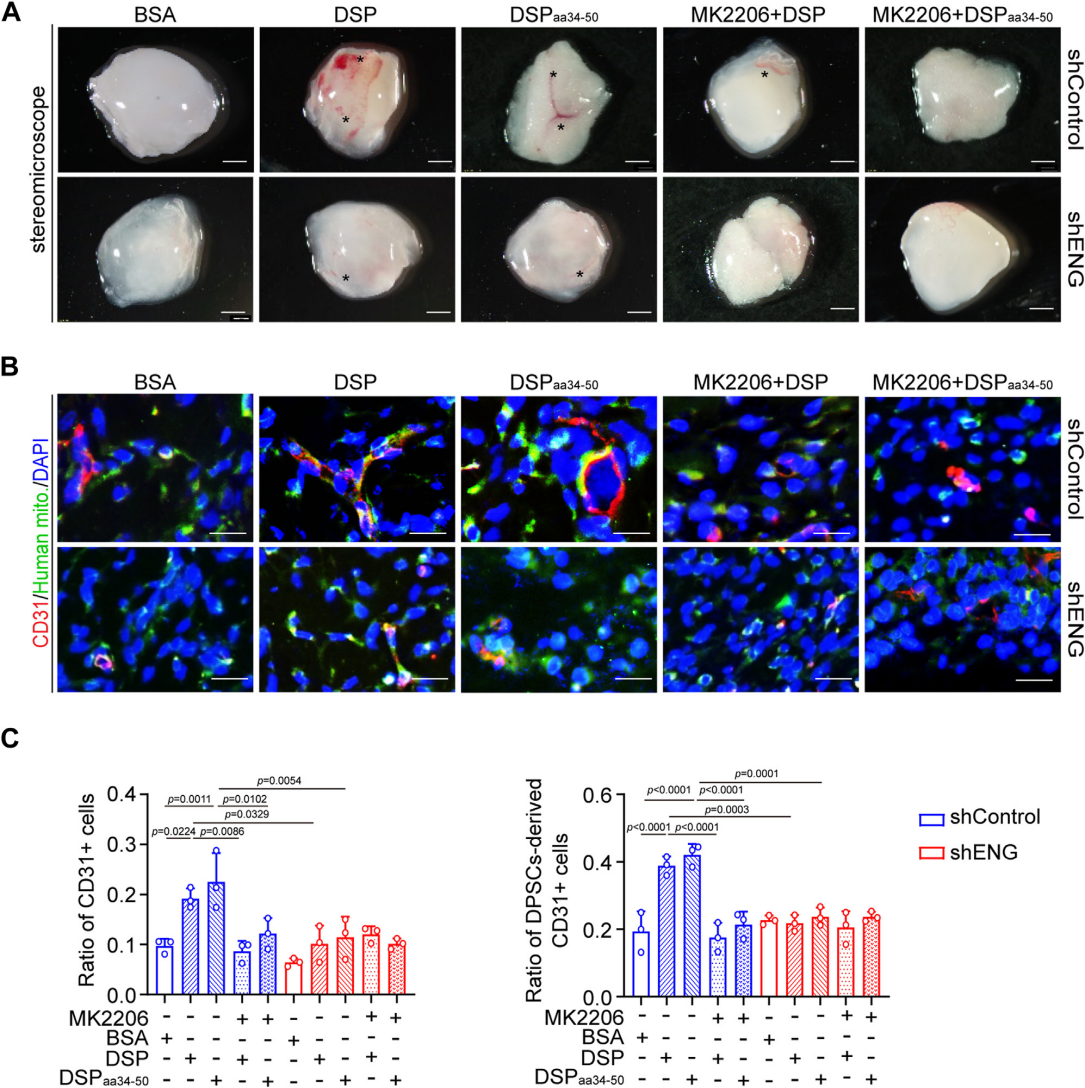
**AKT1 mediates the endothelial differentiation of DPSCs initiated by DSP and DSP**_**aa34–50**_***ex vivo*.** DPSCs with or without *ENG* knockdown or pretreated with MK2206 (2 μM) or not followed by treatment with indicated proteins (300 ng/ml). After 7 days, cells were harvested and mixed with Matrigel. The mixtures were injected subcutaneously into the ventral side of mice, and the Matrigel plugs were collected after 7 days. *A*, representative images of Matrigel plugs containing DPSCs with indicated treatment. *Asterisks* show blood vessels (n = 3). *B*, representative immunofluorescent images of CD31 and human mitochondria in Matrigel plugs of (*A*). An antihuman mitochondria antibody was used to mark DPSCs and DPSC-derived cells. *C*, ratios of CD31+ cells *versus* total cells and DPSC-derived CD31+ cells *versus* total CD31+ cells in (*B*) (n = 3). The quantification results are represented as mean ± SD (*C*). Two-way ANOVA with Tukey's *post hoc* test (*C*). Scale bars represent 2 mm for (*A*) and 50 μm for (*B*). DSP, dentin sialoprotein; DPSC, dental pulp stem cell; ENG, endoglin.

In Matrigel angiogenesis assays, MK2206 pretreatment hampered the capability of DSP- and DSP_aa34–50_-treated DPSCs to form capillary-like structures (Fig. 7, *E* and *F*). As expected, increased blood vessel formation and contributions of DPSCs to vessel assembly in Matrigel plugs were also abolished by AKT1 inhibition shown by immunofluorescent staining (Fig. 8). In the aforementioned experiments, AKT1 inhibition showed a comparative effect to *ENG* knockdown (Figs. 7 and 8). All these results demonstrate that DSP_aa34–50_ promotes the phosphorylation of AKT1 dependent on ENG. The activated AKT1 mediates the migration and endothelial differentiation of DPSCs triggered by DSP_aa34–50_–ENG association.

## Discussion

**Figure 9:**
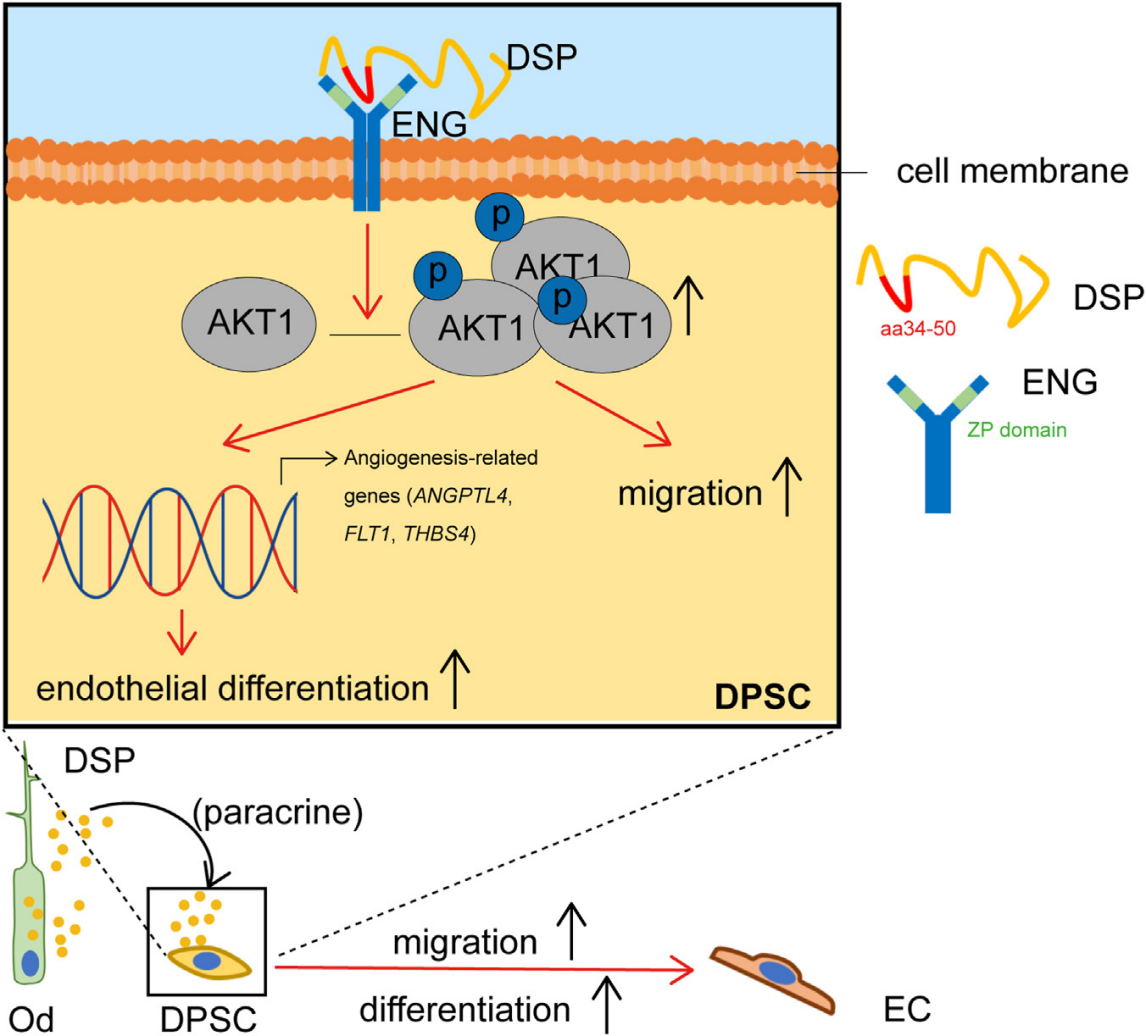
**The working model showing a positive role of DSP in the migration and endothelial differentiation of DPSCs through DSP**_**aa34–50**_**–ENG_ZP_ association and subsequent activation of AKT1 signaling pathway.** DSP, dentin sialoprotein; DPSC, dental pulp stem cell; ENG, endoglin.

In the present study, our findings identify that the amino acid residues 34–50 of DSP and the ZP domain of ENG are responsible for their association. Like DSP, DSP_aa34–50_ promotes the migration and endothelial differentiation of DPSCs dependent on ENG, but DSP without aa34–50 fails to do so. Further experiments show that DSP_aa34–50_–ENG induces the phosphorylation of AKT1 and the expression of angiogenesis-related genes. AKT1 inhibition impairs the molecular and cellular functions of DSP and DSP_aa34–50_. Therefore, DSP_aa34–50_–ENG association causes activation of AKT1 signaling, thus promoting the migration and endothelial differentiation of DPSCs (Fig. 9).

In addition to ENG, DSP interacts with Ocln and integrin β6 according to our previous studies (10, 11). The domains of DSP binding to Ocln and integrin β6 are DSP_aa363–458_ and DSP_aa183–219_, respectively (10, 11). To define the functional fragment of DSP interacting with ENG, we constructed plasmids encoding different truncated DSPs. These data support the notion that DSP_aa34–50_ can be co-immunoprecipitated by ENG and exhibits the same functions as the full-length DSP. Different from triggering odontoblast differentiation and mineralization by DSP_aa363–458_–Ocln and DSP_aa183–219_–integrin β6 interaction (10, 11), our present study reveals that DSP_aa34–50_–ENG association enhances the endothelial differentiation of DPSCs. Therefore, different domains of DSP play distinct roles by interacting with different receptors. A previous study indicates that the ZP domain of betaglycan, a receptor sharing a similar structure to ENG, is important for ligand binding (38). Consistently, we uncovered the involvement of the ZP domain of ENG in DSP_aa34–50_–ENG association. Our present work identifies that DSP functions in an ENG-dependent way.

Many signaling pathways, like Wnt/β-catenin and ERK signaling, have been reported to be involved in endothelial differentiation of DPSCs (21, 22). Although previous studies have reported that ENG functions as a co-receptor in transforming growth factor beta (TGF-β) family (39, 40, 41), TGF-β signaling has been reported to be negatively associated with endothelial differentiation of stem cells from human exfoliated deciduous teeth (SHED), a tooth-derived stem cell possessing similar multilineage differentiation potentials like DPSCs (42, 43). Consistently, the alteration of TGF-β signaling was not observed in our RNA-Seq results.

Because of the essential roles of PI3K–AKT signaling in the migration and differentiation of stem cells (29, 30, 31, 32) and the enrichment of this signaling in the DSP-treated cells shown by our RNA-Seq data, we focused on PI3K–AKT signaling in the following experiments. Our work revealed that AKT1 signaling was significantly upregulated in DSP- or DSP_aa34–50_-treated DPSCs, which was inhibited by *ENG* knockdown. Meanwhile, AKT1 inhibition hampered the migration and endothelial differentiation of DPSCs triggered by DSP and DSP_aa34–50_, confirming that AKT1 signaling mediates the effects of DSP and DSP_aa34–50_. It has been shown that PI3K–AKT signaling pathway regulates the migration of MSCs and endothelial differentiation of cardiac and adipose-derived stem cells (29, 30, 31, 32). Moreover, a previous study has also uncovered that the activation of AKT signaling induces the endothelial differentiation of DPSCs (23), whereas AKT inhibition promotes dental stem cell osteogenic/odontogenic differentiation (44, 45). Collectively, these lines of evidence indicate the positive role of PI3K–AKT signaling in the differentiation of stem cells into endothelial cells, which is also supported by our results. In addition, some other signaling pathways are also enriched in our sequencing data, and additional experiments are needed to uncover whether these pathways are also involved in the migration and endothelial differentiation of DPSCs triggered by DSP.

Collectively, DSP_aa34–50_ interacts with the ZP domain of ENG and consequently activates the AKT1 signaling pathway. Either *ENG* knockdown or AKT1 inhibition suppresses DSP- and DSP_aa34–50_-initiated migration and endothelial differentiation of DPSCs. Therefore, the DSP_aa34–50_–ENG–ATK1 axis promotes the migration and endothelial differentiation of DPSCs. Considering that ENG acts as an accessory receptor in TGF-β receptor family (39, 40, 41), whether other co-receptors participate in the DSP–ENG association and are involved in the effects of DSP awaits further investigations. Moreover, although the applications of anti-DSP and anti-ENG antibodies in immunofluorescence staining were not validated by others, we consider our immunofluorescence staining to be credible because of the negative results of the isotype controls.

## Experimental procedures

### Mice

The animal experiments were approved by the Ethics Committee of Center for Animal Experiment, Wuhan University (protocol no.: WP202110569). Six-week-old BALB/c nude mice were purchased from Hunan Slac Laboratory Animal Co Ltd and maintained at the Center for Animal Experiments/Animal Biosafety Level-III Laboratory (Wuhan University). Mice were housed in ventilated cages in the specific pathogen-free facility with a temperature and light regulated.

### Cell isolation and culture

This study was approved by the Ethics Committee of School of Stomatology, Wuhan University, China (2021-B64) and followed the principles outlined in the Declaration of Helsinki. DPSCs were isolated as previously described (46). In brief, the third molars of 18- to 22-year-old patients were extracted. After washes using PBS (HyClone; catalog no.: SH30256.FS) containing 5% penicillin/streptomycin (P/S) (HyClone; catalog no.: SV30010), the dental pulp tissue was collected and cut into tiny pieces. Trypsin protease (HyClone; catalog no.: SH30042.01) was used to digest pulp tissue pieces at 37 °C for 1 h. Then, cells were filtered, centrifugated, seeded on cell culture flasks, and cultured in minimum essential medium alpha (HyClone; catalog no.: SH30265.01) with 10% fetal bovine serum (VivaCell SCIENCES; catalog no.: C04400) and 1% P/S (HyClone; catalog no.: SV30010) addition. DPSCs were identified as described in our previous study (46). HEK293T cells were cultured in Dulbecco's minimum essential medium (HyClone; catalog no.: SH30243.FS) supplemented with 10% fetal bovine serum (tbdscience; catalog no.: TBD11HT) and 1% P/S (HyClone; catalog no.: SV30010). The medium was changed every other day.

To induce the endothelial differentiation of DPSCs, the medium was changed to endothelial medium (Sciencell; catalog no.: 1001).

### Plasmid construction

ENG-HA plasmid (Sinological; catalog no.: HG10149-CY) was purchased from Sino Biological. DSP-GFP, DSP-His, DSP_aa34–50_-His, and DSP_Δ34–50_-His plasmids were constructed by using ClonExpress II One Step Cloning Kit (Vazyme; catalog no.: C112), and the truncation mutant plasmids were constructed by using Mut Express II Fast Mutagenesis Kit V2 (Vazyme; catalog no.: C214) according to the manufacturers' instructions. The primers for plasmid construction are listed in Table S1.

### Plasmid transfection, shRNA virus infection, AKT1 knockdown, and inhibition of AKT signaling

Plasmid transfection was performed in HEK293T cells at 70% confluence using Lipofectamine 2000 (Invitrogen; catalog no.: 11668019) or Lipofectamine 3000 (Invitrogen; catalog no.: L3000015) according to the manufacturers' instructions. After 48 h, the cell lysates were collected and used for following experiments.

To knockdown the expression of ENG, viruses containing shRNA targeting *ENG* or the negative control scramble shRNA were purchased from Genechem. At around 20% confluence, cells were infected with viral supernatants supplemented with 1 μg/ml polybrene (Yeason; catalog no.: 40804ES76). After 48 h, the cells with virus transfection were selected by 1.5 μg/ml puromycin (Yeason; catalog no.: 54752ES08).

The siRNAs targeting *AKT1* (siAKT1#1 and siAKT1#2) or scramble siRNA were purchased from Genepharma. The following siAKT1 oligos were used: (#1) sense sequence 5′-ACAAGGACGGGCACATTAA-3′ and antisense sequence 5′-UUAAUGUGCCCGUCCUUGUTT-3′; (#2) sense sequence 5′-CAAGGGCACTTTCGGCAAG-3′ and antisense sequence 5′-CUUGCCGAAAGUGCCCUUGTT-3′. For *AKT1* knockdown, DPSCs were seeded into plates. When cells arrived at 70% confluence, siRNA transfection was performed using Lipofectamine 3000 (Invitrogen; catalog no.: L3000015) according to the manufacturer's instruction.

To block AKT1 signaling, cells were pretreated with the specific inhibitor for AKT, MK2206 (2 μM, MedChemExpress; catalog no.: HY-108232).

### Recombinant protein purification

To purify recombinant proteins, the pet42b-DSP, pet42b-DSP_aa34–50_, and pet42b-DSP_Δ34–50_ plasmids were constructed and transformed into BL21 (DE3)-competent cells (Vazyme; catalog no.: C504-02). Expressions of proteins were induced with 1 mM IPTG (Beyotime; catalog no.: ST1416-5g) at 25 °C overnight. Recombinant DSP proteins were purified using Ni–NTA Beads (Smart-Lifescience; catalog no.: SA004005) according to the manufacturer's instruction. Verifications of these proteins were carried out with Coomassie Blue SuperFast Staining Solution (Instant Version) (Beyotime; catalog no.: P0003S) and WB assays using anti-DSP (1:100 dilution; Novus; catalog no.: NBP2-92546) and anti-His (1:100 dilution; Abcam; catalog no.: ab9108) antibodies.

### Co-immunoprecipitation experiments

Overexpression plasmids were transfected into HEK293T cells. After 48 h, cells were rinsed with PBS and lysed using NP-40 lysis buffer (50 mM Tris–HCl [pH 7.4], 150 mM NaCl, and 1% NP-40) (Beyotime; catalog no.: P0013F) containing protease inhibitor cocktail (MedChemExpress; catalog no.: HY-K0010) for 10 min at 4 °C. The extracted lysates were collected and centrifugated at 11,340*g* for 10 min. The supernatants were harvested, and the following procedures were performed as described previously (47). In brief, the supernatants were divided into Input (10%), IP (immunoprecipitation [45%]), and immunoglobulin G (IgG) (45%) groups. The primary antibodies against HA (2 μg, Abclonal; catalog no.: AE008), GFP (2 μg, Abclonal; catalog no.: AE011), or the isotype IgG (2 μg, Beyotime; catalog no.: A7028/A7016) were mixed with the supernatants, and the mixtures were then rotated at 4 °C overnight. The next day, protein A/G magnetic beads (Bimake; catalog no.: B23202) were mixed with the samples and rotated gently at room temperature for 1 h. After washes using NP-40 lysis buffer (50 mM Tris–HCl [pH 7.4], 150 mM NaCl, and 1% NP-40) (Beyotime; catalog no.: P0013F) for five times, the supernatants were discarded, and the beads were resuspended with 1× SDS (Biosharp; catalog no.: BL502B). The collected samples were denatured at 95°C for 10 min and analyzed by WB experiments.

### Cell immunofluorescent staining

HEK293T cells with ENG overexpression were incubated with recombinant DSP, DSP_aa34–50_, or DSP_Δ34–50_ protein (0.5 μg/ml) at 37 °C for 1 h. Then, the samples were fixed with 4% paraformaldehyde (Biosharp; catalog no.: BL539A) at room temperature for 5 min, washed with PBS for three times, and blocked using 5% BSA (BioFroxx; catalog no.: 4240GR100) for 1 h at 37 °C. Next, the rabbit anti-6X His tag (1:100 dilution, Abcam; catalog no.: ab9108) and goat anti-ENG (1:40 dilution, R&D Systems; catalog no.: AF1320) antibodies were incubated at 4 °C overnight. Alexa Fluor Red 594 Donkey anti-Rabbit IgG (1:200 dilution, Antgene; catalog no.: ANT030) and Alexa Fluor Red 488 Donkey anti-Goat IgG (1:80 dilution, Antgene; catalog no.: ANT025) antibodies were used as the secondary antibodies and incubated at 37 °C for 1 h. Finally, the samples were mounted by Antifade Mounting Medium with 4',6-diamidino-2-phenylindole (DAPI) (Beyotime; catalog no.: P0131-5mL), and images were captured using OLYMPUS BX53 fluorescence microscope (OLYMPUS Corporation).

### *In situ* PLA assay

*In situ* PLA assays were performed using the Duolink kit (Sigma–Aldrich, DUO92003/DUO92005/DUO92008). HEK293T cells with ENG-HA overexpression were incubated with recombinant DSP, DSP_aa34–50_, or DSP_Δ34–50_ protein (0.5 μg/ml) at 37 °C for 1 h. Cells were then fixed using 4% paraformaldehyde (Biosharp; catalog no.: BL539A) at room temperature for 5 min, washed using PBS, and blocked using blocking solution (Sigma–Aldrich; DUO92003) at 37 °C for 1 h. The rabbit anti-6X His tag polyclonal antibody (1:100 dilution, Abcam; catalog no.: ab9108) and goat anti-ENG polyclonal antibody (1:40 dilution, R&D Systems; catalog no.: AF1320) were added and incubated at 4 °C overnight. Afterward, ligation of PLA probes and amplification of PLA signals were performed according to the manufacturer's instruction. The samples were mounted with Antifade Mounting Medium with DAPI (Beyotime; catalog no.: P0131-5mL), and the images were captured by OLYMPUS BX53 fluorescence microscope.

To determine the DSP–ENG association *in vivo*, 5-μm slices of postnatal 2 mouse molars were deparaffinized, rehydrated, antigen retrieved, and blocked using blocking solution (Sigma–Aldrich) at 37 °C for 1 h. The rabbit anti-DSP polyclonal antibody (1:100 dilution, Novus; catalog no.: NBP2-92546) and goat anti-ENG polyclonal antibody (1:40 dilution, R&D Systems; catalog no.: AF1320), or the rabbit IgG (1:100 dilution, Beyotime; catalog no.: A7016) and goat IgG (1:40 dilution, Beyotime; catalog no.: A7007) were incubated at 4 °C overnight. The following procedures were carried out according to the manufacturer's instruction, and the images were also captured by OLYMPUS BX53 fluorescence microscope under the same conditions as the isotype controls.

### Molecular docking analysis

Protein–protein docking between DSP_aa34–50_ and ENG_ZP_ was performed using HDOCK (http://hdock.phys.hust.edu.cn/). The protein structural domain of ENG_ZP_ was obtained from the Protein Data Bank database (https://www.rcsb.org/). Protein structural domain of DSP_aa34–50_ was predicted using AlphaFold (version 2.3.2; Deepmind), and PyMOL (version 2.1; Schrödinger) was used to investigate DSP_aa34–50_–ENG_ZP_ interaction and visual analysis.

### Scratch wound healing assay

The scratch wound assays were used to evaluate the migratory capacity of DPSCs. DPSCs with or without *ENG* knockdown were seeded onto 6-well plates. After being cultured to confluence, cells were scratched by a 200 μl pipette, and the medium was then changed to serum-free medium with supplemented BSA, DSP, DSP_aa34–50_, or DSP_Δ34–50_ (300 ng/ml) with MK2206 (2 μM, MedChemExpress; catalog no.: HY-108232) pretreatment or not. The images were collected at 0 and 24 h after scratch-making using OLYMPUS LIB80 microscope (OLYMPUS Corporation), and the migration areas were calculated.

### RT–qPCR analysis

Total RNAs from cells were harvested and reversely transcribed to complementary DNAs using the ABScript III RT Master Mix for qPCR with gDNA Remover (Abclonal; catalog no.: RK20433). Hieff qPCR SYBR Green Master Mix (No Rox) (Yeason; catalog no.: 11201ES08) was used to perform RT–qPCR analysis. The primer sequences are listed in Table S2. *ACTB* was used for normalization. The delta–delta Ct method was used for RT–qPCR data analysis.

### WB analysis

Total proteins were harvested using NP-40 lysis buffer (50 mM Tris–HCl [pH 7.4], 150 mM NaCl, and 1% NP-40) (Beyotime; catalog no.: P0013F) containing 1/100 protease inhibitor Cocktail (MedChemExpress; catalog no.: HY-K0010) at 4 °C for 10 min. Then the samples were lysed supersonically and centrifugated at 4 °C with 11,340*g* for 10 min. The supernatants were harvested, mixed with 5× SDS-PAGE Protein Sampling Buffer (Biosharp; catalog no.: BL502B), and denatured at 95 °C for 10 min. Next, protein samples were fractionated by 8% or 10% SDS-PAGE and transferred to Trans-blot membranes (Roche; catalog no.: 03010040001). The membranes were blocked by 5% skimmed milk (Beyotime; catalog no.: P0216-300g) at room temperature for 2 h and incubated with the primary antibodies at 4 °C overnight as follows: VEGFR2 (1:1000 dilution, Abclonal; catalog no.: A11127), CD31 (1:5000 dilution, Novus; catalog no.: NBP1-71663), ACTIN (1:8000 dilution; Abclonal, catalog no.: AC038), GFP (1:2000 dilution, Abclonal; catalog no.: AE011), HA (1:5000 dilution, Abclonal; catalog no.: AE008), ENG (1:1000 dilution, Proteintech; catalog no.: 10862-1-AP), p-AKT1 (1:1000 dilution, Immunoway; catalog no.: YP0590), AKT1 (1:1000 dilution, Abclonal; catalog no.: A17909). After washes with Tris-buffered saline with Tween-20 for three times, the membranes were incubated with secondary antibody (1:6000 dilution, Merck; catalog no.: A9044/A0545) and detected by WesternBright ECL HRP substrate (Advansta; catalog no.: K-12045).

### Matrigel angiogenesis assay

The 24-well plates after precooling were coated with 200 μl growth factor–reduced Matrigel matrix (Corning; catalog no.: 354230) and incubated at 37 °C for 45 min. DPSCs with *ENG* knockdown or pretreated with MK2206 (2 μM, MedChemExpress; catalog no.: HY-108232) were cultured with supplement of BSA, DSP, DSP_aa34–50_, or DSP_Δ34–50_ protein (300 ng/ml) for 7 days. Then cells were harvested and seeded on Matrigel at a density of 180,000 cells per well. After incubation in endothelial medium at 37 °C for 14 h, CellTrace carboxyfluorescein succinimidyl ester dye (2 μM, Invitrogen; catalog no.: C34570) was used, and the vascular-like structures were photographed using OLYMPUS LIB80 microscope. The total lengths, the number of nodes, and the number of junctions were calculated and evaluated using ImageJ software (NIH).

### *Ex vivo* Matrigel plug assay

Six-week-old BALB/c nude mice were anesthetized with 2% isoflurane. DPSCs with different treatment for 7 days were collected, mixed with Ceturegel Matrix Phenol Red-Free, LDEV-Free Matrigel (Yeason; catalog no.: 40186ES08), and the mixture was then injected subcutaneously into the ventral side of mice in a final volume of 400 μl. The Matrigel plugs were harvested after 7 days, and the collected samples were captured immediately. After image capture, the plugs were fixed using 4% paraformaldehyde fix solution (Biosharp; catalog no.: BL539A) at 4 °C overnight. The samples were dehydrated and then embedded with paraffin. The 5-μm-slices of samples were performed for the following histological analysis.

### Immunofluorescent staining

For histological staining, 5-μm slices were deparaffinized, rehydrated, and blocked with 5% BSA at 37 °C for 1 h. Primary anti-CD31 (1:200 dilution, Servicebio; catalog no.: GB12063), antimitochondria (1:800 dilution, Abcam; catalog no.: ab92824), the rabbit IgG (1:100 dilution, Beyotime; catalog no.: A7016), goat IgG (1:40 dilution, Beyotime; catalog no.: A7007), anti-DSP (1:100 dilution, Novus; catalog no.: NBP2-92546), and/or anti-ENG (1:40 dilution, R&D Systems; catalog no.: AF1320) antibodies were incubated with slices at 4 °C overnight. After washes for three times, the secondary antibodies, Alexa Fluor Red 594 Donkey anti-Rabbit IgG (1:200 dilution, Antgene; catalog no.: ANT030), Alexa Fluor Red 488 Donkey anti-Mouse IgG (1:200 dilution, Antgene; catalog no.: ANT023), and/or Alexa Fluor Red 488 Donkey anti-Goat IgG (1:80 dilution, Antgene; cataog no.: ANT025) were incubated at 37 °C for 1 h. Next, the slices were mounted using Antifade Mounting Medium with DAPI (Beyotime; catalog no.: P0131-5mL), and images were captured using OLYMPUS BX53 fluorescence microscope under the same conditions as the isotype controls.

The analysis of codistribution between DSP and ENG immunofluorescence signals was carried out using ImageJ software (NIH, Bethesda, USA).

### Statistical analyses

The differences between two groups were analyzed by using Student's *t* test. Among three or more groups, data were analyzed by one-way or two-way ANOVA followed by Tukey's *post hoc* test. *p* < 0.05 was considered statistically significant. Experiments were repeated at least three times. All results are represented as mean ± SD. We used GraphPad Prism software (version 8.0; GraphPad Software, Inc) to analyze all data statistically.

## Data availability

All RNA-Seq datasets are available through the National Center Biotechnology Information databases (PRJNA1218007). All data in this study are presented in the article and the supporting information.

## Supporting information

This article contains supporting information (Figs. S1, S2, Tables S1, and S2).

## Conflict of interest

The authors declare that they have no conflicts of interest with the contents of this article.

## Supporting information

Supporting Information

Catalogue Numbers of all reagents
