# Supplementary material for: Dentin sialoprotein promotes endothelial differentiation of dental pulp stem cells through DSPaa34–50–endoglin–AKT1 axis
Source: J Biol Chem. 2025 Mar 4;301(4):108380. doi: 10.1016/j.jbc.2025.108380 (PMC11997338; doi:10.1016/j.jbc.2025.108380)
Supplement: Supporting Information [file mmc1.pdf]

Supporting Information for "DSP promotes endothelial differentiation of dental pulp stem cells through $DSP_{aa34\text{-}50}$-endoglin-AKT1 axis"

Authors:
Ximin Xu[1, 2, 3], Jing Fu[1, 2, 3], Guobin Yang[1], Zhi Chen[1], Shuo Chen[4], Guohua Yuan[1, 2, 3, *]

Affiliation:
[1]State Key Laboratory of Oral & Maxillofacial Reconstruction and Regeneration, Key Laboratory of Oral Biomedicine Ministry of Education, Hubei Key Laboratory of Stomatology, School & Hospital of Stomatology, Wuhan University, Wuhan, Hubei, China;
[2]Frontier Science Center for Immunology and Metabolism, Wuhan University, Wuhan, China;
[3]Hubei Provincial Key Laboratory of Developmentally Originated Disease, Wuhan, Hubei, China;
[4]Department of Developmental Dentistry, School of Dentistry, The University of Texas Health Science Center at San Antonio, San Antonio, TX, United States.
[*]Corresponding author: yuanguohua@whu.edu.cn

Keywords: Dentin sialoprotein, endothelial differentiation, endoglin, dental pulp stem cells

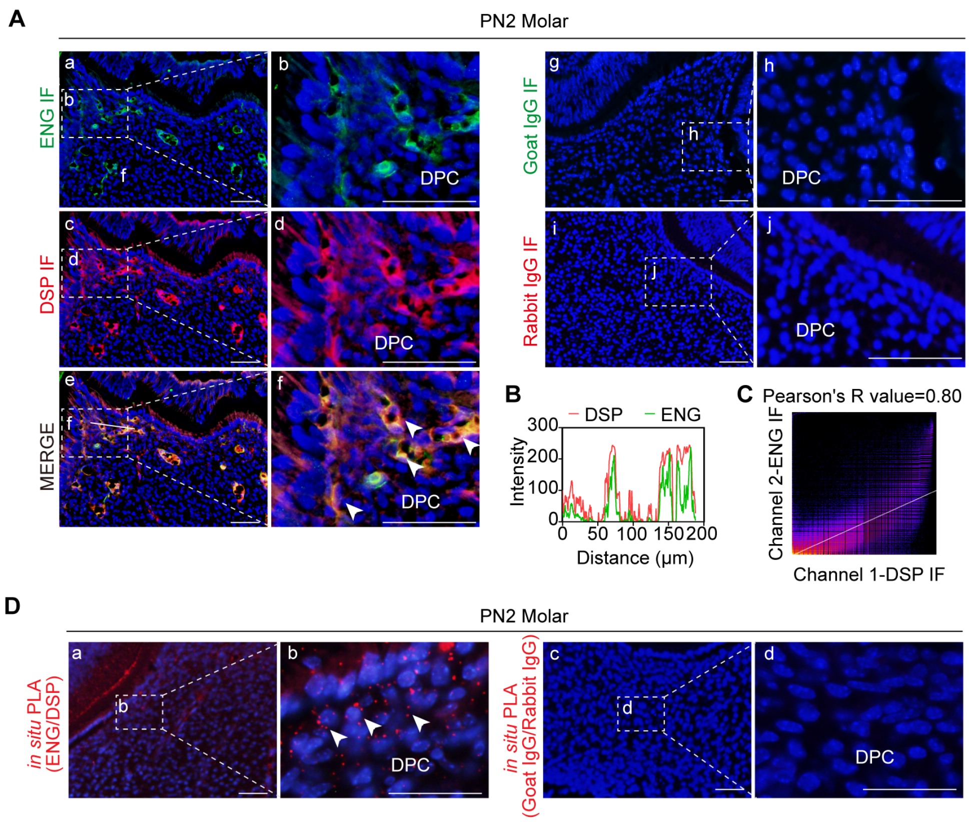

Supplementary Figure S1. Immunofluorescent (IF) staining and *in situ* proximity ligation (PLA) assay of DSP and ENG in postnatal (PN) 2 mouse molars.

A, representative immunofluorescent images of non-immune goat immunoglobulin G (IgG), non-immune rabbit IgG, DSP, and ENG in PN2 mouse molars. b, d, f, h and j are higher magnifications of the corresponding rectangles in a, c, e, g and i, respectively. Arrows point to the co-distribution signals for DSP and ENG. Non-immune goat IgG and non-immune rabbit IgG were used as isotype controls for ENG and DSP antibodies, respectively.

B, plot of immunofluorescent intensity of DSP and ENG along the white line in *A* (e).

C, plot of quantitative analysis of co-distribution between DSP and ENG in *A* (e).

D, representative images showed the DSP-ENG PLA signals in PN2 mouse molars. b and d are higher magnifications of the corresponding rectangles in a and c. Arrows point to the positive signals for DSP-ENG PLA signals. Non-immune goat IgG and non-immune rabbit IgG were used as isotype controls for ENG and DSP antibodies, respectively.

Scale bars, 50 μm for *A* (a-j) and *D* (a-d). DPC, dental papilla cell.

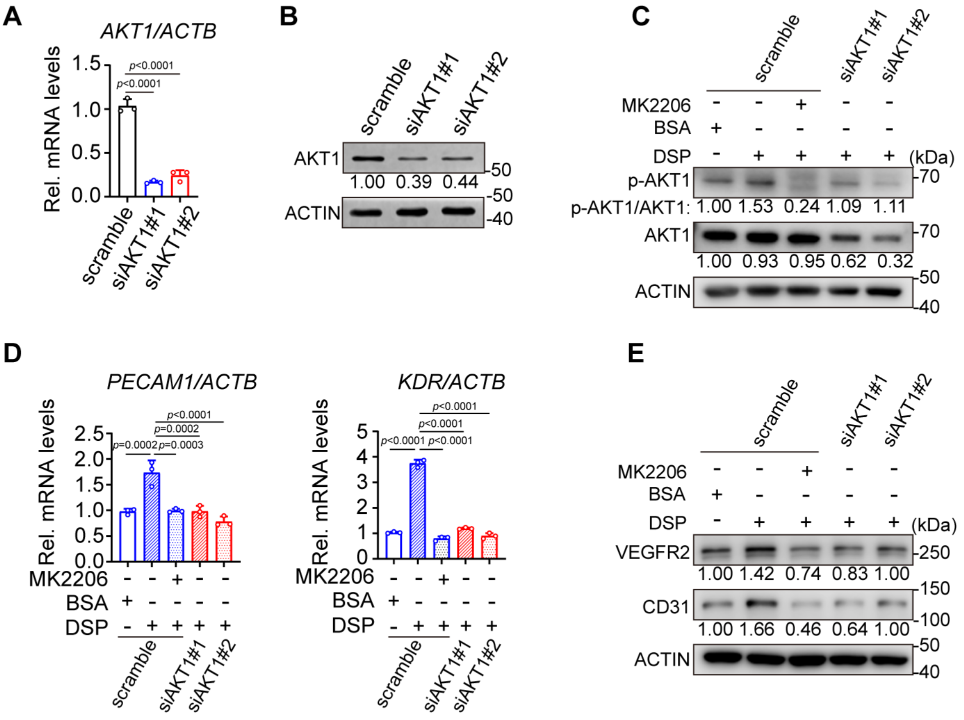

Supplementary Figure 2. AKT1 inhibition using MK2206 showed the equivalent effects to *AKT1* knockdown with small interfering RNAs (siRNAs).

A and *B*, verification of the efficiency of *AKT1* knockdown in DPSCs at mRNA (*A*) and protein (*B*) levels using RT-qPCR and WB analysis. *ACTB* was used for normalization. The Delta-Delta Ct method was used to analyze data (*A*). ACTIN served as loading control, and the relative ratios of AKT1 vs. ACTIN were shown (*B*). Each experiment was performed 3 times.

C-E, DPSCs transfected with *AKT1* siRNAs (siAKT1#1 or siAKT1#2) or pretreated with MK2206 (2 μM, an inhibitor targeting AKT signaling pathway) were cultured with indicated proteins (300 ng/mL). WB analysis showed the levels of p-AKT1 and AKT1 in DPSCs with indicated treatment. ACTIN served as a loading control, and relative ratios of p-AKT1 vs. AKT1 and AKT1 vs. ACTIN were shown (*C*). The mRNA levels of *PECAM1* and *KDR* in DPSCs with indicated treatment for 7 days were assessed by RT-qPCR (n = 3). *ACTB* was used for normalization. The Delta-Delta Ct method was used to analyze data (*D*). The protein levels of CD31 and VEGFR2 in DPSCs with indicated treatment for 7 days were detected using WB analysis. ACTIN served as a loading control, and relative ratios of VEGFR2 vs. ACTIN and CD31 vs. ACTIN were shown (*E*).

The quantification results are represented as mean ± SD (*A* and *D*). One-way ANOVA with Tukey's post hoc test for (*A*) and two-way ANOVA with Tukey's post hoc test for (*D*).

Supporting Information Table 1. Sequences of primers used for plasmid construction.

Primer name	Sequence (5'-3')
D1-F	AAGCTGGCTAGCAACGAAGAGGGGAATATTGAGGG
D1-R	TTCGTTGCTAGCCAGCTTGGGTCTCCCTATAGT
D2-F	TGGCTAGCGAGAATTCCTGTAGAAACGAGGGTA
D2-R	AGGAATTCTCGCTAGCCAGCTTGGGTCTCCCTA
D3-F	GCTGGCTAGCAGTAAAGAATATTATGACCCTGAAGGC
D3-R	TCTTTACTGCTAGCCAGCTTGGGTCTCCCTATA
D4-F	CTGGCTAGCGATAAAGGACAACATGGAATGATCTT
D4-R	TCCTTTATCGCTAGCCAGCTTGGGTCTCCCTAT
1-100-F	CATTAGCAGGTACCATGGTGAGCAAGGGCGAGG
1-100-R	CCATGGTACCTGCTAATGTGGAATATGTAGAAAAACTC
f1-F	TAAATGCCGGTACCATGGTGAGCAAGGGCGAGG
f1-R	CCATGGTACCGGCATTTAACTCATCCTGTACTGACA
f2-F	CTAGCAGTGGAACCATCAAAGAAAGTGGTGTCC
f2-R	TGATGGTTCCACTGCTAGCCAGCTTGGGTCTCC
f3-F	CTGGCTAGCATTCCAGTTCCTCAAAGCAAACCA
f3-R	AACTGGAATGCTAGCCAGCTTGGGTCTCCCTAT
f4-F	CCATGAATGGTACCATGGTGAGCAAGGGCGAGG
f4-R	CCATGGTACCATTCATGGATTTTTCGACATGTCTC
Frag-F	AAGCTGGCTAGCTTGCATCTCCTAGCAAGATCAAAT
Frag-R	ATGCAAGCTAGCCAGCTTGGGTCTCCCTATAGT
Δ34-50-F	TCCATGAATAGTGGAACCATCAAAGAAAGTGGT
Δ34-50-R	GGTTCCACTATTCATGGATTTTTCGACATGTCTC
hEngZP1-F	AAGCTTGGTACCCCCGCACCGATCCAGACCACT
hEngZP1-R	TGCGGGGGTACCAAGCTTGGTGGCGGCCCCTAT
hEngZP2-F	TGGGGGTGGAGGCTCTTATCCTTACGACGTGCC
hEngZP2-R	TAAGAGCCTCCACCCCCACCAGACAGGTCAGGGCTGAT
hEngOR1-F	CTTGGTACCGAAACAGTCCATTGTGACCTTCAG
hEngOR1-R	GACTGTTTCGGTACCAAGCTTGGTGGCGGCCCC
hEngOR2-F	TAGGCTGCAGACCTCAGGGGGTGGAGGCTCTTATCC
hEngOR2-R	CCTGAGGTCTGCAGCCTACCACCGCAGCTGGAG
hEngΔZP-F	AGACCTCATGCACAAGCAAAGGCCTCGTCCTGC
hEngΔZP-R	TGCTTGTGCATGAGGTCTGCAGCCTACCACCGC

Supporting Information Table 2. The primers used for RT-qPCR.

Primer name	Sequence (5’-3’)
PECAM1-F	CCCAGCCCAGGATTTCTTAT
PECAM1-R	ACCGCAGGATCATTTGAGTT
KDR-F	GTGATCGGAAATGACACTGGAG
KDR-R	CATGTTGGTCACTAACAGAAGCA
ACTB-F	ATTGCCGACAGGATGCAGA
ACTB-R	GAGTACTTGCGCTCAGGAGGA
ANGPTL4-F	CAGCAACTCTTCCACAAGGT
ANGPTL4-R	GTTCACCAAAAATGGCGGAG
CXCL8-F	CTTGGCAGCCTTCCTGATTT
CXCL8-R	ATTCTCAGCCCTCTTCAAAAACT
FLT1-F	GAAAACGCATAATCTGGGACAGT
FLT1-R	GCGTGGTGTGCTTATTTGGA
HMOX1-F	ATAGAAGAGGCCAAGACTGC
HMOX1-R	GCATAAAGCCCTACAGCAAC
IL1A-F	AGATGCCTGAGATACCCAAAACC
IL1A-R	CCAAGCACACCCAGTAGTCT
LEP-F	CCAGGATCAATGACATTTCACA
LEP-R	TGGATAAGGTCAGGATGGGG
THBS4-F	TGATGATGATGATGACAATGATGG
THBS4-R	TGACCTCTGCGTTCTCTGG
